# Association of separate components of the metabolic syndrome and suicidal risk in patients with schizophrenia

**DOI:** 10.1192/j.eurpsy.2021.1398

**Published:** 2021-08-13

**Authors:** V. Gerasimova, E. Kornetova, A. Kornetov, I. Mednova, N. Kornetov

**Affiliations:** 1 Department Of Endogenous Disorders, Mental Health Research Institute, Tomsk National Research Medical Center, Russian Academy of Sciences, Tomsk, Russian Federation; 2 Department Of Fundamental Psychology And Behavioral Medicine, Siberian State Medical University, Tomsk, Russian Federation; 3 Laboratory Of Molecular Genetics And Biochemistry, Mental Health Research Institute, Tomsk National Research Medical Center, Russian Academy of Sciences, Tomsk, Russian Federation; 4 Department Of Psychiatry, Addictology And Psychotherapy, Siberian State Medical University, Tomsk, Russian Federation

**Keywords:** suicide risk, schizophrénia, Metabolic syndrome, obesity

## Abstract

**Introduction:**

Patients with schizophrenia have increased cardiovascular and suicide risk. Metabolic syndrome (MetS) is widespread in this group, however, there are no unambiguous data on the relationship between the separate components of metabolic syndrome and suicide risk.

**Objectives:**

To examine the relationship between the separate components of the MetS and suicide risk in patients with schizophrenia.

**Methods:**

We examined 64 patients with schizophrenia. All patients received antipsychotic therapy in doses comparable in chlorpromazine equivalents. We measured serum levels of lipids, glucose and insulin. The visceral fat level was determined through the non-invasive bioimpedance analysis with an “Omron BF508” scale and body composition monitor. Suicide risk was assessed using Beck Hopelessness Inventory. There were identified two groups of examined: with MetS and without MetS. In both groups were distinguished two subgroups: patients with normal range of hopelessness and patients with mild and moderate hopelessness. Subgroups were compared among themselves for a number of anthropometric, biochemical and clinical indicators. Statistical analysis was conducted using Mann-Whitney U-test. Reliability level corresponded to p<0.05. This study was supported by a grant from the Russian Science Foundation 18-15-00011.

**Results:**

**Figure:**
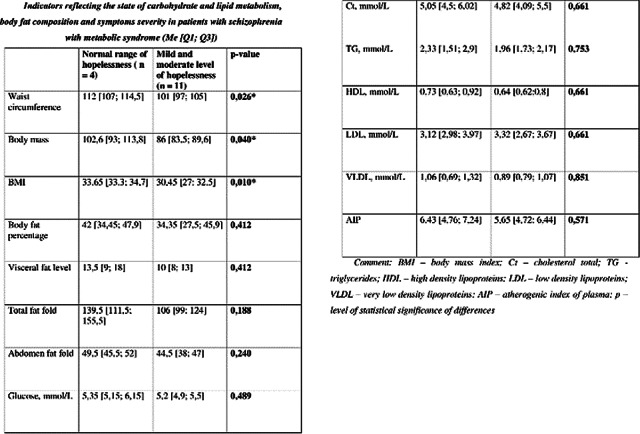

Waist circumference, body weight and BMI in subgroup with normal hopelessness range in the group of patients with MetS were significantly higher (figure 1).

**Conclusions:**

We were able to establish a negative relationship between the waist circumference, body weight and BMI with suicide risk in schizophrenia patients. It can be assumed that adipose tissue can play a “protective” role in the suicidal behavior of schizophrenia patients.

